# Bulk Segregant Analysis Reveals Genomic Regions Associated With Imidacloprid Resistance in the Colorado Potato Beetle

**DOI:** 10.1002/ece3.72527

**Published:** 2025-11-18

**Authors:** Alitha Edison, Nijat Nariman, Pablo Duchen, Shuqing Xu

**Affiliations:** ^1^ Institute for Evolution and Biodiversity University of Münster Münster Germany; ^2^ Institute of Organismic and Molecular Evolution (iomE) University of Mainz Mainz Germany; ^3^ Institute for Quantitative and Computational Biosciences (IQCB) University of Mainz Mainz Germany

**Keywords:** genetic basis of resistance, insecticide resistance, *Leptinotarsa decemlineata*, neonicotinoid, pesticide, QTL mapping, RNA interference

## Abstract

Given the increased accessibility of genomic techniques, the speed of evolution of resistance, and the large number of genes involved in resistance, investigations into the genetic basis of resistance in more species are pertinent. Despite being an important agricultural pest, only a limited number of genetic mapping studies based on crossed populations have been performed to identify genes involved in resistance in the Colorado potato beetle (CPB, *Leptinotarsa decemlineata* Say). Here, we performed bulk segregant analysis on a mapping population generated by creating advanced intercross lines from five European strains of CPB. We identified eight peaks across chromosomes 1, 8, 10, and 16 involved in resistance against the neonicotinoid, imidacloprid. We identified 337 genes within these peaks and shortlisted three candidates based on gene expression and functional annotation. Among the three candidates identified, we found that an ABC transporter and a galactosyl transferase are expressed in relatively higher amounts in a relatively more susceptible strain than in a resistant strain. We attempted to validate the role of these two genes in insecticide resistance by knocking them down in a resistant strain using RNA interference (RNAi) and performing toxicity experiments. Contrary to our expectations, we found that the activation of the RNAi machinery itself reduced imidacloprid resistance, and the effect is not specific to the tested candidate genes. This raises concern about the suitability of using RNAi for validating insecticide resistance mechanisms in CPB.

## Graphical abstract

Genomic regions potentially associated with resistance against imidacloprid in the Colorado potato beetle were identified here using bulked segregant analysis. Candidate genes were then narrowed down using the functional annotation and expression analysis. RNAi on two genes of interest revealed inconclusive roles for the specific genes in resistance but raised important concerns for the use of GFP as a control in RNAi‐based functional validation of genes.

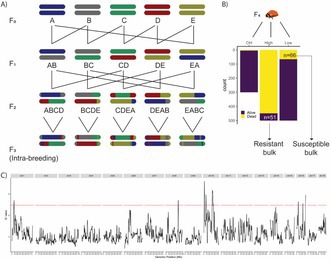

## 1 Introduction

Insecticide resistance is a classic example of natural selection and a global economic problem threatening sustainable food production (Hawkins et al. 2019). Understanding the genetic basis of resistance is crucial for both academic research and applied pest management. This is especially relevant in the current environment where research on genetics‐ and genomics‐based pest management is rapidly advancing (Li et al. 2022; Palli 2014; Yan, Aumann, et al. 2023).

Several target site‐associated genes and metabolic genes are involved in insecticide resistance. Mutations in conventional target sites, including neurotransmitter receptors and ligand‐ and voltage‐gated ion channels, have been shown to increase resistance (ffrench‐Constant et al. 1993; Mutero et al. 1994; Williamson et al. 1996). Among genes that are involved in essential physiological functions like xenobiotic detoxification, transport and excretion in insects, the most ubiquitous resistance‐associated candidates are cytochrome P450 monooxygenases (CYPs), esterases (ESTs), ATP‐binding cassette transporters (ABCs) and glutathione S‐transferases (GSTs) (Amezian et al. 2024; Enayati et al. 2005; Hemingway 2000; Liu et al. 2015; Nauen et al. 2022; Pavlidi et al. 2018). In addition to mutations in target sites and metabolic genes, mutations causing upregulation and downregulation of genes through both *cis‐* and *trans‐*regulatory processes have also been shown to be involved with resistance. Novel mechanisms (Chen, Jiang, et al. 2023; Pu and Chung 2024) are being discovered, possibly faster than ever, owing to technical advances in genetic mapping and reduced sequencing costs.

Colorado potato beetles (CPB, *Leptinotarsa decemlineata* Say) are highly destructive leaf‐eating pests of potato ( *Solanum tuberosum* ). CPB is currently resistant to more than 50 different types of insecticides like DDT, imidacloprid, thiamethoxam and spinosad (Mota‐Sanchez and Wise 2022). Resistance has even been reported against the latest RNA‐interference (RNAi) based pesticide in CPB (Mishra et al. 2021). CPB is an emerging model organism for rapid host adaptation and insecticide resistance research (Alyokhin and Chen 2017; Casagrande 1987; Chen, Cohen, et al. 2023). The known mechanisms of insecticide resistance in CPB are target site insensitivity, and altered detoxification, transport, penetration, and excretion of insecticides (Alyokhin et al. 2008, 2022). Reduced cholinesterase activity is involved with carbofuran resistance (Ioannidis et al. 1992; Wierenga and Hollingworth 1994). Increased levels of monooxygenase enzymes (Rose and Brindley 1985) and esterases (Argentine et al. 1989) are associated with resistance against carbamate and organophosphate insecticides. Mutations typically associated with organophosphate and pyrethroid resistance, namely, the S291G of acetylcholinesterase and L1014F of sodium channel respectively, are present in several resistant populations (Clark et al. 2001; Lee et al. 2000; Malekmohammadi et al. 2012; Tebbe et al. 2016). Hawthorne et al. identified three quantitative genetic loci (QTL) contributing to resistance against a pyrethroid through linkage mapping (Hawthorne 2003).

Neonicotinoids are one of the most used insecticide classes in the world (Bass et al. 2015). Imidacloprid is a neonicotinoid that acts as an inhibitor of the neurotransmitter acetylcholine, causing paralysis and eventually death in insects (Bai et al. 1991). Although imidacloprid has long been a mainstay for managing CPB, the field efficacy of imidacloprid has steadily waned due to beetle populations evolving resistance rapidly. Imidacloprid and two other neonicotinoids are banned in some places, like the EU, but the use of imidacloprid persists elsewhere. Researchers have shown the involvement of several CPB genes in imidacloprid resistance using RNAi. For example, the overexpression of a cytochrome P450 monooxygenase and a uridine diphosphate (UDP) glycosyltransferase gene is associated with imidacloprid resistance (Kaplanoglu et al. 2017).

RNAi is a classic, powerful genomics tool used to decode gene function. The efficiency of RNAi machinery in silencing genes is affected by several factors like the stability of the double stranded RNA (dsRNA), the mode of cellular uptake and the components of the core RNAi machinery (Cooper et al. 2019; Koo and Palli 2024). Although RNAi is efficient in Coleopterans in general and CPB especially (Baum et al. 2007; Palli 2014; Zhu et al. 2011), results can vary somewhat between populations (Mehlhorn et al. 2020).

To our knowledge, cross‐based genetic mapping of imidacloprid resistance has not previously been performed in CPB. Here, we aimed to investigate the genetic basis of imidacloprid resistance in CPB using the cost‐effective mapping technique called bulked segregant analysis (BSA) in which individuals showing extreme phenotypes for a trait (very high or very low trait values) are pooled into two separate bulks (Kurlovs et al. 2019; Li and Xu 2022; Michelmore et al. 1991; Van Leeuwen et al. 2012; Yu et al. 2021). The key questions we ask in this study are: (1) What are the genes involved in imidacloprid resistance in CPB? (2) Are any of those genes differentially expressed in the resistant genotype compared to the susceptible genotype? (3) Does the knockdown of any of the differentially expressed genes affect the susceptibility levels of CPB against imidacloprid?

## 2 Materials and Methods

### 2.1 Insects, Plants and Insecticide

**FIGURE 1:**
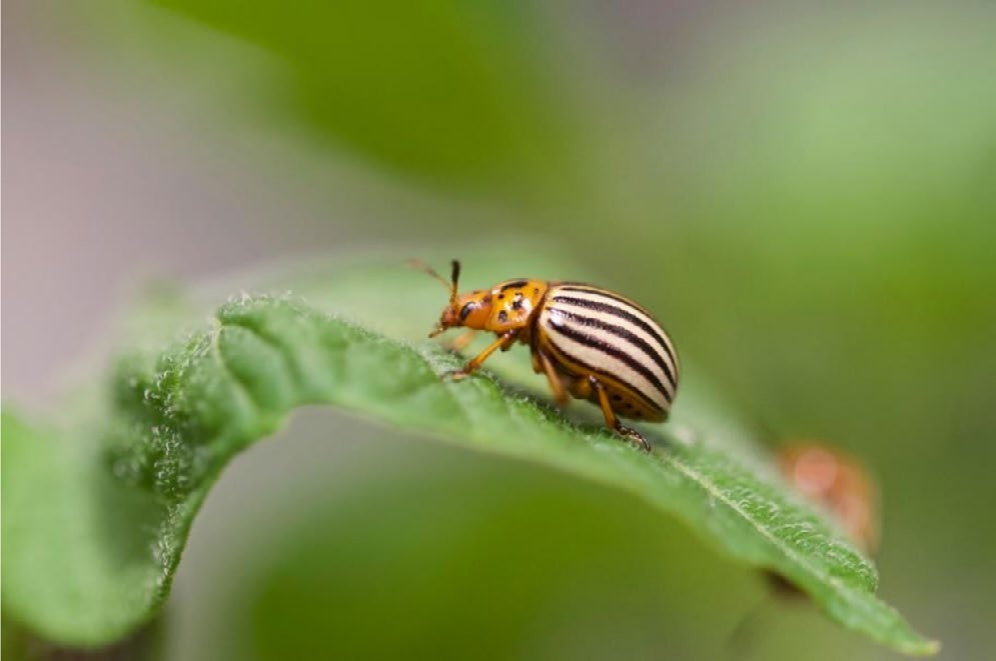
The study system. The Colorado potato beetle (CPB) is a highly destructive pest of potato plants. These beetles are resistant to over 50 different insecticides.

Colorado potato beetles (Figure 1) were obtained from Dr. Ralf Nauen's lab, Bayer AG (Monheim, Germany). The five original strains were collected by the supplier and team from different parts of Europe at different time points (Mehlhorn et al. 2020). They were namely, D01 (Germany, 2002), E01 (Spain, 2014), E02 (Spain, 2017), E06 (Spain, 2012), and U01 (Ukraine, 2012). The E06 strain was the most resistant out of the five with a resistance ratio of 25.03‐fold against the most susceptible D01 strain (Edison et al. 2024).

All insects were reared on five‐week‐old potato ( *Solanum tuberosum* ) plants (Annabelle variety, seed potatoes purchased from Ellenberg's Kartoffelvielfalt GmbH & Co. KG, Barum, Germany) under insecticide‐free conditions for at least 20 generations in 85 cm × 45 cm × 55 cm‐sized insect cages. The beetle rearing, plant growing, and experiments were carried out in a greenhouse (Methods sections 2.2 and 2.3) and a climate chamber (Methods section 2.7) under a long day photoperiod (16:8 L:D) at a temperature of 24°C. The detailed rearing method is stated elsewhere (Edison et al. 2024).

Imidacloprid, a neonicotinoid insecticide, was used in our experiments in the form of water‐dispersible granules available commercially by the name Confidor WG 70 (70 g/kg Imidacloprid, Bayer AG, Monheim, Germany). The insecticide granules were mixed in water and applied to the soil. Due to the discontinuation of Confidor in the EU, technical grade imidacloprid (Bayer AG, Monheim, Germany) was used for the RNAi experiments.

### 2.2 Generating the Segregating Population

**FIGURE 2:**
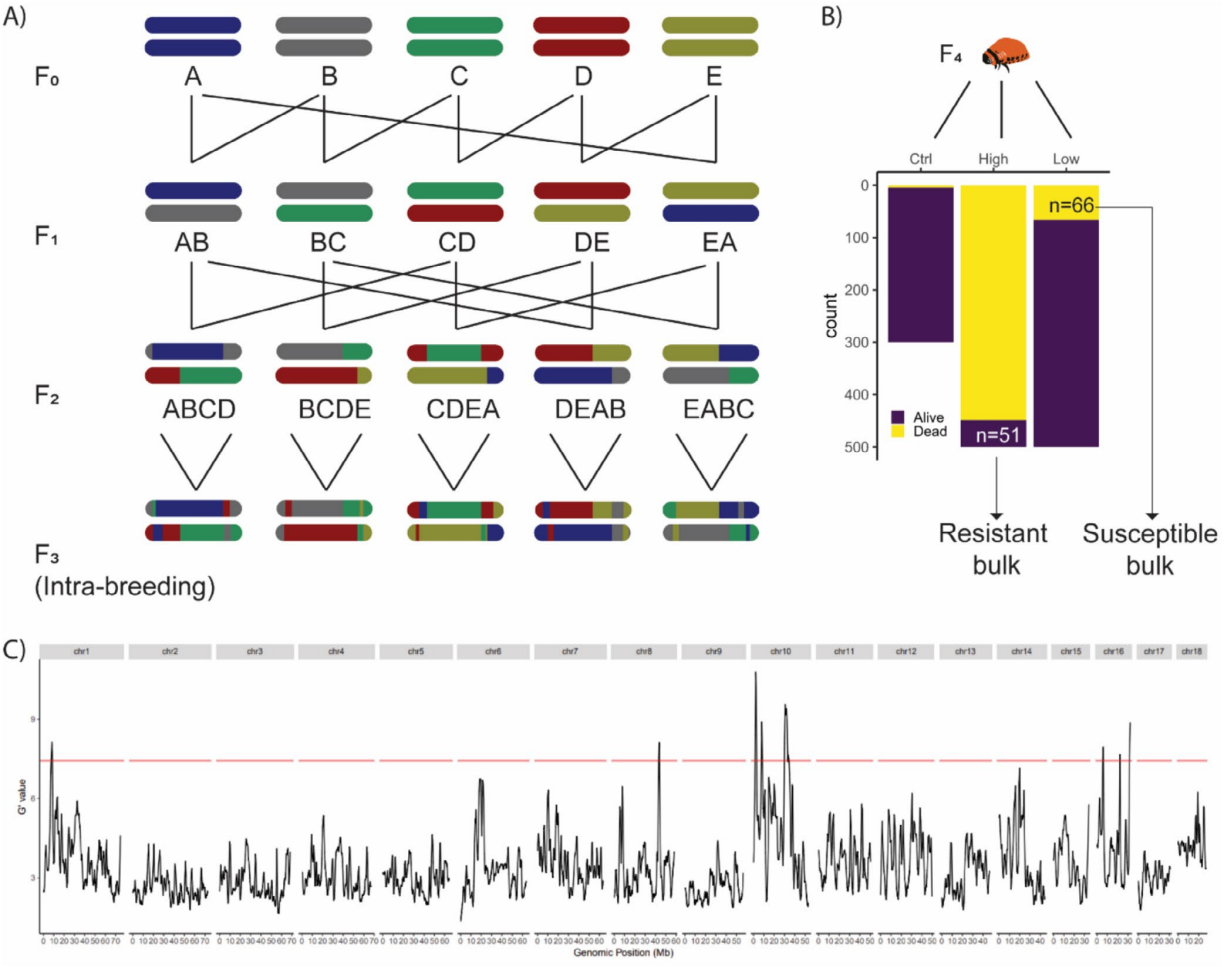
Eight QTL peaks associated with imidacloprid resistance identified through Bulk Segregant Analysis. (A) Crossing scheme. Five strains of Colorado potato beetle (CPB) were intercrossed to generate a genetically diverse mapping population for QTL mapping via bulked segregant analysis. The letters denote the parental strains: A = D01, B = E01, C = E02, D = E06 and E = U01. For each combination of intercrosses shown here, male x female and female × male crosses were constructed. This resulted in 10 crosses at the F1 stage and 20 crosses at the F2 stage. At the F3 stage, each cross was allowed to inbreed. (B) Phenotyping assays. The inverted bar plot shows the number of dead (yellow) and alive (purple) larvae in the three treatments of the phenotyping assay—(1) Ctrl: water, (2) high: 250 ppm imidacloprid, (3) low: 1 ppm imidacloprid. The arrows indicate the number and type of bulk (resistant bulk: Alive in the high treatment & susceptible bulk: Dead in the low treatment) the larvae formed for BSA. (C) QTL peaks. The G' statistic is plotted for the whole genome of CPB. The chromosome number is indicated on top. The red horizontal line represents the threshold for significance. The genomic position in Mb is shown on the x‐axis.

The five strains of CPB were intercrossed to result in 20 lines as indicated in the schematic in Figure 2A. For every two strains that were crossed, reciprocal crosses were made with four mating pairs in each combination. This sequential and random crossing method was used to facilitate higher resolution in mapping (Darvasi and Soller 1995).

The 20 lines were allowed to mate and develop for one generation without intermixing to form the F3 generation. The offspring of this inbred generation were further subjected to phenotyping assays. While generating the crosses, for each generation, pupae were sexed (Pelletier 1993), and the males and females were stored in separate plastic boxes. As it was crucial not to overrepresent or underrepresent each strain or cross, sex determination was once again performed on the freshly emerged adults as well. The cages and boxes were well labeled, and insects were handled with utmost care to avoid cross‐contamination.

### 2.3 Phenotyping Assays

An equal number of larvae from each of the final crosses was subjected to phenotyping assays to create pools of individuals showing extreme phenotypes for imidacloprid sensitivity (highly resistant vs. highly susceptible).

Toxicity assays were performed on five‐day‐old F4 2nd instar larvae of the segregating population to separate the individuals into two extreme bulks (bulks of highly susceptible and highly resistant individuals, Figure 2B). Insecticide was applied topically on larvae that were placed in a petri dish lined with filter paper, following the IRAC Susceptibility Test Method 029 (IRAC Methods Working Group 2013). Two aqueous solutions containing either 1 ppm (low‐concentration treatment) or 250 ppm (high‐concentration) imidacloprid were used. These concentrations were chosen based on pretests which showed that at 1 ppm, 10% of the larvae died (highly susceptible), and at 250 ppm, 10% of the larvae survived (highly resistant). It is recommended that the bulks consist of 10%–20% of the individuals tested to ensure sufficient statistical power for further analysis (Magwene et al. 2011). In each of the two insecticide treatments, 500 larvae were tested, and 300 larvae were tested in the control treatment (water without insecticide). 1 μL of the aqueous solution for the respective treatment was applied using a micropipette on the top of each larva. The larvae were supplied with sufficient leaves to feed on throughout the duration of the assay to avoid the effects of starvation. Mortality was assessed after 48 h, and the dead larvae (*n* = 66) were collected from the low‐concentration treatment, and the surviving larvae (*n* = 51) were collected from the high‐concentration treatment to form bulks of highly susceptible and highly resistant individuals, respectively. All larvae were frozen in liquid nitrogen and stored at −20°C until DNA isolation.

### 2.4 DNA Isolation and Whole Genome Sequencing (WGS)

Genomic DNA was isolated from all pooled samples using the Monarch Genomic DNA purification kit #T3010 (New England BioLabs, Frankfurt, Germany) following the manufacturer's protocol. From the toxicity assays, 66 and 51 larvae were pooled to form the susceptible and resistant bulks, respectively. The concentration of DNA was assessed using a Nanodrop 1000 (Thermo Scientific, Hennigsdorf, Germany). Whole genome sequencing was performed using an Illumina Novaseq sequencer at Novogene (Cambridge, UK).

### 2.5 Bulked Segregant Analysis

Read trimming was performed with Skewer (Jiang et al. 2014). Mapping was performed with BWA‐*MEM* (Li 2013) and variant calling was performed using GATK (McKenna et al. 2010). The high‐quality variants were used as markers for the bulked segregant analysis using the G' statistic (Magwene et al. 2011) implemented in the *QTLseqr* package (Mansfeld and Grumet 2018) in R. Candidate genes were examined using the published genome annotation and the functional annotation of CPB (Yan, Zhang, et al. 2023). Using the Gene Expression Atlas (GEA) of CPB (Wilhelm et al. 2025), genes that were not sufficiently expressed were filtered out.

### 2.6 Relative Expression of Candidates in Susceptible and Resistant Genotypes

RT‐qPCR experiments were performed to compare the expression of the candidate genes in a susceptible and a resistant strain. As the highly susceptible D01 strain used for the initial crosses was no longer available, the susceptible E01 strain was used for these experiments. Three 2nd instar larvae each from the susceptible (E01) and resistant (E06) strains were used for RNA extraction, cDNA synthesis, and qPCR. Total RNA was isolated from the whole larval body using the RNeasy Mini Kit (Qiagen, Hilden, Germany) following the manufacturer's protocol. The yield and purity were then measured using the Nanodrop 1000 (Thermo Scientific, Hennigsdorf, Germany). cDNA synthesis was performed with 0.1 μg of total RNA using the RevertAid First‐Strand cDNA synthesis kit (ThermoFisher Scientific, Unna, Germany) with oligo‐dT primers. For qPCR, previously published primer sequences were used for the housekeeping genes RP18 and ARF1 (Shi et al. 2013). For the candidates, primer pairs were designed using PrimerBLAST (Ye et al. 2012). Based on a dilution series (1:10, 1:100 and 1:1000), primer efficiencies were calculated for each primer pair used. The sequences and efficiencies of the chosen primers are shown in Table S1. Then, for performing RT‐qPCR, a 1:100 dilution of the cDNA was used. RT‐qPCR was performed in a RotorGene Q system (Qiagen) using the KAPA SYBR FAST kit (Roche, Basel, Switzerland). The program consisted of initial denaturation at 98°C for 3 min, followed by 40 cycles of denaturation (98°C, 3 s) and annealing/extension (60°C, 20s). RT‐qPCR data was analyzed following the $2^{-\Delta \Delta C_{T}}$ method (Livak and Schmittgen 2001) with expression normalized to two housekeeping genes, RP18 and ARF1.

### 2.7 Functional Validation Using RNAi

RNAi was performed to silence the expression of the target genes to validate their function. As the goal was to validate if knocking down these genes in a resistant background would decrease resistance, experiments were performed on the resistant E06 strain. 2nd instar larvae weighing 3.5–4.5 mg were allowed to ingest leaf discs coated with 50 ng/12 μL double stranded RNA (dsRNA). Three types of dsRNA, namely, ds*GFP*, ds*LdNA19763*, and ds*LdNA20673* respectively targeting the enhanced Green Fluorescent Protein (eGFP) gene (GenBank: U55761.1), and the CPB genes *LdNA19763* and *LdNA20673* were designed by and purchased from Eupheria Biotech (Dresden, Germany). All dsRNA sequences were formatted using EMBL‐EBI Job Dispatcher sequence analysis tools framework (Madeira et al. 2024) and are given in Table S2. 12 μL of aqueous solutions of ds*LdNA20673* (*n* = 89) or ds*LdNA19763* (*n* = 90) was carefully spread on a 2 cm diameter leaf disc (cut from leaves at the 2nd‐4th position from the top of 5‐weeks‐old plants) using a pipette tip. Water (*n* = 83) was used as the control for evaluating the effects of RNAi machinery, and ds*GFP* (*n* = 88) was used as the control for evaluating gene‐specific functions. Once the leaf discs were dry, they were placed in a petri dish lined with moistened filter paper. A single larva was placed on the leaf disc. The dsRNA treatment was repeated daily with freshly treated leaf discs for 3 days. After that, the larvae were fed with untreated fresh leaves for 2 days. This was done to avoid any priming effects of the dsRNA treatment on the mortality caused by the insecticide. On the 6th day, the larvae from each treatment were assigned to two groups for toxicity assays: 100 ppm of technical grade imidacloprid or acetone (as the control for the toxicity assay). 2 μL insecticide or acetone was applied topically on the larvae. Data were analyzed by performing a binomial generalized linear model (“stats” package; R Core Team 2023) on the counts of ‘dead’ or ‘alive’ larvae as a function of the treatment. Binomial tests of proportions (prop. test, “stats” package; R Core Team 2023) were also performed to identify which proportions are different from each other. To verify the successful knockdown of the genes, three larvae were collected from each treatment and snap‐frozen on the 6th day before the toxicity experiment. RT‐qPCR was then performed as previously described to check the expression of the candidate genes in all the different dsRNA treatments relative to the control (water) samples. RT‐qPCR data was analyzed following the $2^{-\Delta \Delta C_{T}}$ method (Livak and Schmittgen 2001) with expression normalized to two housekeeping genes, RP18 and ARF1. The figures were created using the “ggplot2” (Wickham 2016) and “viridis” (Garnier et al. 2023) packages. All analyses were performed using R version 4.3 (R Core Team 2023) and RStudio version 2024.09.0 (Posit team 2024).

## 3 Results

### 3.1 Eight QTL Peaks Governing Imidacloprid Resistance Identified Using BSA

Toxicity assays performed on the F4 offspring of the intercrossed lines (Figure 2A) at the 2nd instar larval stage yielded approximately 10% of the larvae in each of the bulks (Figure 2B). Figure 2C shows the G' statistic (Magwene et al. 2011) calculated using the R package QTLseqr (Mansfeld and Grumet 2018) for the genome. We identified eight significant QTL peaks involved with imidacloprid resistance in CPB in chromosomes 1, 8, 10 and 16 (Figure 2C). In total, 171,711 SNPs overlapped the significant regions.

### 3.2 Candidate Genes Narrowed Down Based on the Gene Expression Atlas and the Functional Annotation

Using the genome annotation of CPB, 337 genes were identified in the eight peaks. A full list of those 337 genes along with information about their potential function is given in Table S2. According to the GEA of CPB (Wilhelm et al. 2025), 126 genes were sufficiently expressed (> 5 Transcripts Per Million (TPM)) in the first, second or third instar larvae. Among them, SNPs predicted to result in at least one nonsynonymous amino acid change were present in 65 genes using SNPEff (Cingolani et al. 2012) (Table 1, Figure S1). We then manually searched the functional annotation for genes commonly associated with insecticide resistance including cytochrome P450 monooxygenases (CYPs), ATP‐binding cassette (ABC) transporters and glutathione S‐transferases (GSTs). Two ABC transporters (*LdNA19763*, *LdNA19944*) and one galactosyl transferase (*LdNA20673*) were found on the 10th chromosome. The expression profiles of these three genes across the first, second and third larval instars were downloaded from the GEA website (Wilhelm et al. 2025) (Figure S2).

**TABLE 1:** List of 65 candidate genes. A list of genes that are sufficiently expressed in at least one larval stage of the Colorado potato beetle (Wilhelm et al. 2025) and additionally contain SNPs that result in at least one amino acid change. The potential function of the gene is given in the ‘BLAST Annotation’ column if available; ‘—’ indicates a lack of information. The ‘Start’ and ‘End’ columns indicate the position of the gene in bp within the specific chromosome.

Sl. no.	Chr	Start	End	Gene	BLAST Annotation
1	1	7541214	7541843	LdNA_787	Slit homolog 3 protein
2	1	7829263	7852833	LdNA_795	Zinc carboxypeptidase A 1
3	1	7859085	7876267	LdNA_797	NEDD8‐activating enzyme E1 regulatory subunit
4	1	7869091	7870339	LdNA_798	Enoyl‐CoA delta isomerase 1, mitochondrial
5	1	7876684	7879733	LdNA_799	Bifunctional purine biosynthesis protein ATIC
6	1	7879380	7894610	LdNA_800	Prostaglandin F synthase 1
7	1	7910994	7911822	LdNA_802	—
8	1	7955891	7976740	LdNA_803	—
9	1	8003077	8005511	LdNA_805	Cathepsin L‐like proteinase
10	1	8041483	8045590	LdNA_808	Cathepsin L‐like proteinase
11	1	8112169	8116310	LdNA_812	Crustapain
12	1	8221306	8274067	LdNA_816	Probable chitinase 10
13	1	8333172	8352457	LdNA_818	—
14	1	8491027	8499576	LdNA_827	—
15	1	8722199	8730280	LdNA_843	Gastrula zinc finger protein XlCGF57.1 (Fragment)
16	1	8734421	8750822	LdNA_844	—
17	10	1698756	1700628	LdNA_19747	Syntaxin‐6
18	10	1713214	1719534	LdNA_19748	Small ribosomal subunit protein uS7
19	10	1725625	1732713	LdNA_19749	—
20	10	2254329	2313006	LdNA_19763	ATP‐dependent translocase ABCB1
21	10	2323030	2343199	LdNA_19764	Ubiquitin carboxyl‐terminal hydrolase 5
22	10	2349139	2360544	LdNA_19765	Lysophosphatidylserine lipase ABHD12
23	10	2364307	2370090	LdNA_19766	—
24	10	2409450	2423753	LdNA_19769	Inositol oxygenase
25	10	2439301	2503688	LdNA_19770	Putative helicase mov‐10‐B.1
26	10	2571028	2571447	LdNA_19771	Lysozyme C‐3
27	10	2682578	2684445	LdNA_19776	Probable transaldolase
28	10	2803033	2920459	LdNA_19778	Rho guanine nucleotide exchange factor 12
29	10	7485132	7515401	LdNA_19917	Lactosylceramide 1,3‐N‐acetyl‐beta‐D‐glucosaminyltransferase
30	10	7683600	7817876	LdNA_19922	G‐protein coupled receptor Mth2
31	10	7734254	7734793	LdNA_19923	—
32	10	7779199	7779717	LdNA_19925	—
33	10	7792849	7801228	LdNA_19926	—
34	10	7847991	7848506	LdNA_19931	—
35	10	7865598	7895072	LdNA_19933	G‐protein coupled receptor Mth2
36	10	7899320	7916084	LdNA_19935	Probable ATP‐dependent RNA helicase DDX52
37	10	7917349	7918317	LdNA_19936	Snurportin‐1
38	10	7922417	7931189	LdNA_19937	Aspartate–tRNA ligase, cytoplasmic
39	10	7934588	7944525	LdNA_19939	Protein IWS1 homolog
40	10	8033082	8034442	LdNA_19943	UDP‐glucuronic acid decarboxylase 1
41	10	8130421	8200857	LdNA_19944	ATP‐dependent translocase ABCB1
42	10	8220396	8221244	LdNA_19947	Probable E3 ubiquitin‐protein ligase sinah
43	10	8237205	8241177	LdNA_19948	—
44	10	8261655	8311566	LdNA_19949	Probable G‐protein coupled receptor Mth‐like 3
45	10	8320277	8359394	LdNA_19951	—
46	10	30286861	30288083	LdNA_20612	Facilitated trehalose transporter Tret1‐2 homolog
47	10	31122297	31268581	LdNA_20633	Protein spitz
48	10	31496992	31503626	LdNA_20638	Probable ribosome production factor 1
49	10	31510400	31526539	LdNA_20639	Retinoblastoma‐binding protein 5 homolog
50	10	31526591	31538840	LdNA_20640	Mitochondrial import receptor subunit TOM40 homolog 1
51	10	31559226	31710217	LdNA_20642	Ras‐GEF domain‐containing family member 1B‐A
52	10	31953391	31954257	LdNA_20645	Distal membrane‐arm assembly complex protein 2
53	10	31958730	32100639	LdNA_20646	Maternal embryonic leucine zipper kinase
54	10	31975273	31983719	LdNA_20647	Cysteine‐rich hydrophobic domain‐containing protein 2
55	10	32877990	32882425	LdNA_20667	—
56	10	32893983	32932389	LdNA_20668	—
57	10	33146685	33180110	LdNA_20672	Protein transport protein Sec24C
58	10	33182649	33194725	LdNA_20673	Beta‐1,4‐galactosyltransferase 1
59	10	33196991	33203422	LdNA_20674	—
60	10	33228756	33229043	LdNA_20675	—
61	10	34061079	34069751	LdNA_20690	—
62	16	5836460	5968027	LdNA_30474	DENN domain‐containing protein 2B
63	16	6052497	6187195	LdNA_30479	Fibrillin‐1
64	16	22159527	22181417	LdNA_31050	Phenoloxidase 1
65	16	32275644	32278200	LdNA_31557	—

### 3.3 Relative Expression of Candidates in the Susceptible and Resistant Strains

**FIGURE 3:**
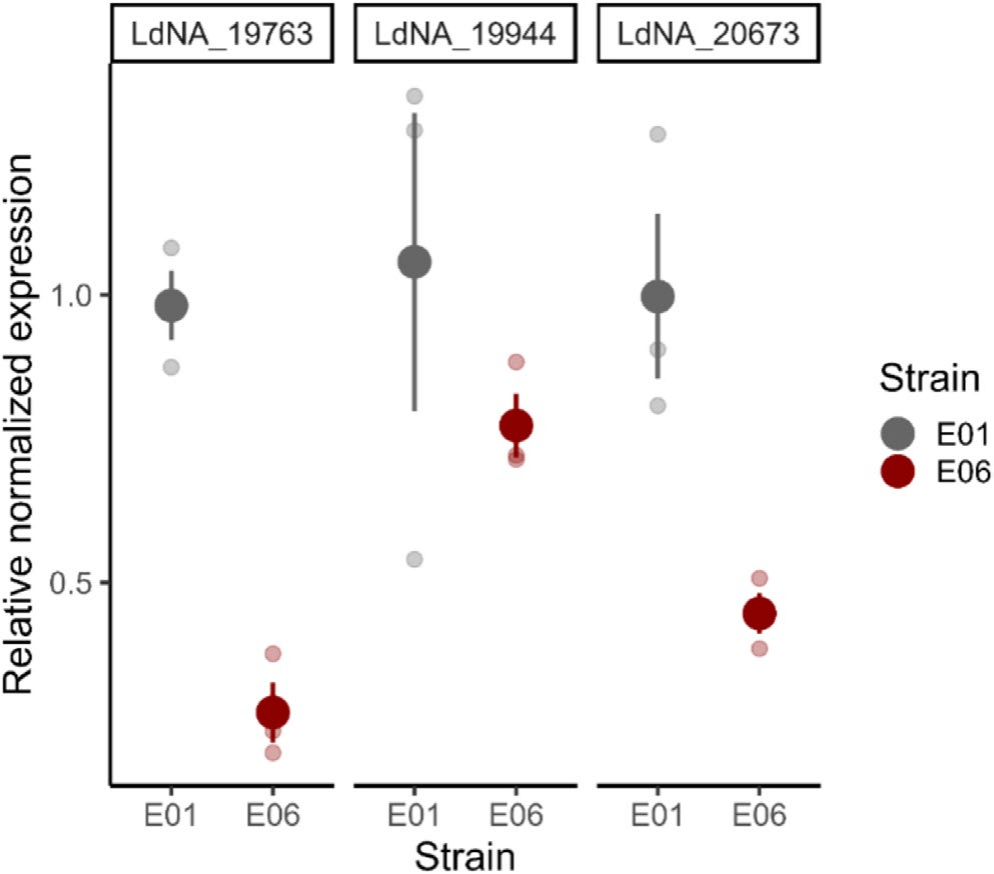
Relative expression of three candidate genes in two Colorado potato beetle strains. The graph shows the relative expression of three candidate genes (*LdNA_19763*, *LdNA_19944* and *LdNA_20673*). The colors indicate the two strains (Gray: E01 and Red: E06). The expression was calculated relative to the comparatively more susceptible strain, E01. The opaque dots represent the mean values while the smaller faded dots indicate the values for the three replicates. The error bars show the standard error of the mean.

We quantified the expression differences of the candidate genes between a resistant and a susceptible strain. Two out of the three candidates (*LdNA19763* and *LdNA20673*) were expressed in higher amounts in the relatively more susceptible E01 strain compared to the relatively more resistant E06 strain (*LdNA19763*: *p* < 0.001, *LdNA19944*: *p* = 0.387, *LdNA20673*: *p* = 0.055, *t*‐test, Figure 3). The expressions of *LdNA19763* and *LdNA20673* were elevated 3.6‐ and 2.2‐fold respectively in the E01 strain.

### 3.4 Functional Validation of the Candidates

**FIGURE 4:**
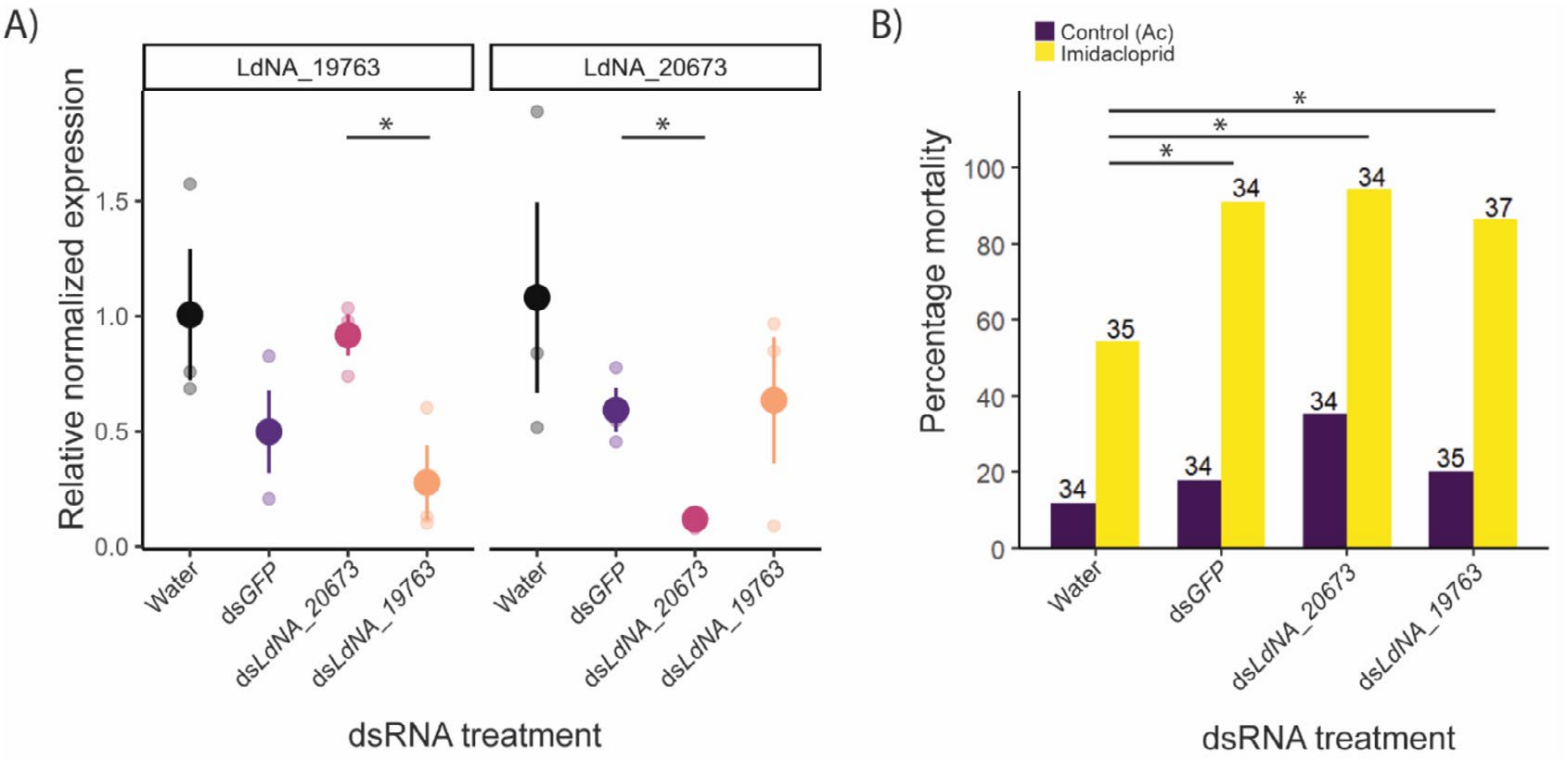
Functional validation of two candidates using RNAi. (A) Verification of knockdown after RNAi. The graph shows the relative expression of the two knocked‐down genes (*LdNA19763* and *LdNA20673*) in the different dsRNA treatments (*x*‐axis). Asterisks indicate significant differences in expression. The expression was calculated relative to the water control. The opaque dots show the mean values, while the smaller faded dots show the values for the three replicates. The error bars show the standard error of the mean. (B) Mortality after insecticide treatment. The bar plot shows the percentage mortality after insecticide and control treatments for each of the different dsRNA treatment groups (Water, dsGFP, ds*LdNA20673* and ds*LdNA19763*). The colors indicate the two treatments in the toxicity assay (Purple: Control (acetone) and Yellow: Imidacloprid). The numbers above the bars are the number of larvae tested in each group. Asterisks indicate significant differences in mortality.

The RT‐qPCR data comparing the expressions of the ABC transporter *LdNA19763* and the galactosyl transferase *LdNA20673* among different RNAi treatments showed high variation, and the knockdowns were partially successful (Figure 4A). Out of six pairwise comparisons of gene expression, two showed statistically significant differences. Expression of gene *LdNA20673* differed significantly between ds*GFP* and ds*LdNA20673* treatments (*p* = 0.03, *t*‐test). Expression of gene *LdNA19763* differed between ds*LdNA19763* and ds*LdNA20673* treatments (*p* = 0.04, *t*‐test). The expression levels of the targeted genes were not significantly different from the negative water control.

After RNAi and toxicity assays, the final mortality was affected by the dsRNA treatment type (*p* < 0.001, GLM: Status~Treatment, family = binomial, Figure 4B). Mortality due to the insecticide treatment was significantly lower in the water treatment than in the other treatments (*p* < 0.001, binomial test of proportions) while the control (acetone) mortality was not different between the treatments (*p* = 0.1, binomial test of proportions). The mortality after the knockdown of genes *LdNA19763* or *LdNA20673* was not different from that in the ds*GFP* treatment (*p* = 0.5, binomial test of proportions). Due to the difference between the two negative controls, we did not find any conclusive evidence for the involvement of these two genes in resistance against imidacloprid in CPB.

## 4 Discussion

In this study, we attempted to identify the genetic basis of resistance against imidacloprid in the Colorado potato beetle by intercrossing five strains of beetles and performing bulked segregant analysis. We identified eight QTL peaks associated with resistance in which an ABC transporter gene and a galactosyl transferase gene were expressed in higher amounts in the susceptible strain when compared to the resistant strain. We then performed functional validation of the two genes using RNAi. Our results did not provide conclusive evidence for the involvement of these two specific genes in imidacloprid resistance, a finding we attribute to the general effect of RNAi activation on beetle physiology.

While manually searching for genes that have some functional relevance in resistance, we found three ABC transporters belonging to the B subfamily in the peaks. The role of ABC transporters in insecticide resistance in CPB is not as clear as in other insects like *Drosophila* spp. or *Tribolium* spp. Due to their natural excretory functions and the similarity of many insecticides to the substrates of ABC transporters, they have been implicated in insecticide resistance (Amezian et al. 2024; Dermauw and Van Leeuwen 2014; Gott et al. 2017). Gaddelapati et al. (2018) showed that the knockdown of two ABCH transporters increased the susceptibility of CPB to imidacloprid. In contrast, Güney et al. (2021) found that the knockdown of an ABCB1 transporter made larvae more resistant to Cry toxins. At the same time, the knockdown of an ABCG transporter did not affect susceptibility levels of CPB against imidacloprid (Kaplanoglu et al. 2017). Similarly, an ABCB transporter had no effects on the resistance of CPB against ivermectin (Favell et al. 2020). Further studies on the molecular functions of the ABC transporters in CPB might help understand the detailed mechanism behind the involvement of these enzymes in insecticide transport and resistance.

Other potential candidates include five chemoreceptors that we found in the peak in chromosome 1. Chemoreceptor genes have been found to be overexpressed in some resistant insect populations (Li et al. 2023; Xu et al. 2022). Chemosensory proteins and other sensory receptor genes were initially implicated in resistance due to their potential role in altering behavior. Additionally, due to their versatility in transporting and sequestering several types of compounds, chemosensory proteins are also garnering attention as a novel mechanism of resistance (Pelosi et al. 2018; Pu and Chung 2024).

Numerous previous studies have shown the involvement of CYP genes in insecticide resistance across several insect species (Daborn et al. 2002; Kaplanoglu et al. 2017; Nauen et al. 2022; Zhu et al. 2016). Notably, no CYPs were found among the 337 genes within the significant QTL peaks (Table S3). CPB possesses about 74–98 CYP genes that carry out different functions including the detoxification of insecticides (Nauen et al. 2022; Wan et al. 2013). Changes in the CYP genes may constitutively increase their expression in resistant populations of several insect species, thus contributing to increased resistance. Additionally, transcription factors like the cap ‘n’ collar isoform C (CncC) have also been shown to regulate the increased expression of CYPs and the subsequent detoxification of insecticides (Gaddelapati et al. 2018; Kalsi and Palli 2017). Lv et al. (2023) have recently demonstrated that the CF2‐II transcription factor negatively regulates the expression of ABC transporters and influences insecticide resistance in cotton aphids. Interestingly, we found nine C2H2 type zinc finger genes in our peaks (Table S3).

The reduced expression of the two candidate genes in the resistant strain compared to the susceptible strain is counterintuitive to a simple model of metabolic resistance. However, such a pattern is not unheard of for many resistance‐associated genes, including ABCs and CYPs (Dively et al. 2020; Guo et al. 2015; Kaplanoglu et al. 2017; Zhu et al. 2016). Reduced activity of ABC transporters that are involved in the delivery of xenobiotics to the target sites combined with continued detoxification by other enzymes could result in decreased efficacy of pesticides. Another possibility is that the transporters at the basolateral lining of the intestine pump the toxins out into the hemolymph resulting in increased absorption (Ming and Thakker 2010). In such cases, a reduced expression of ABC transporters would correlate with increased resistance to toxins (Amezian et al. 2024; Denecke et al. 2022; Wu et al. 2023). It is unclear how such an expression pattern might be regulated in resistant populations. Negative regulatory pathways mediated by transcription factors like CnCC or C2H2 zinc finger proteins may be involved (Lv et al. 2023; Palli 2020). More research must be conducted to further understand the complex ways in which ABC transporters influence the distribution of toxins in the body.

Advances in functional genomic techniques and the vast amount of genomic data have made it possible to investigate the role of specific genes in insecticide resistance in many non‐model pests (Homem and Davies 2018; Przybyla and Gilbert 2022). In our study, we attempted to functionally validate the role of two genes, namely, *LdNA19763* and *LdNA20673*. The expression levels of these two target genes were not significantly different from the negative water treatment which raises the possibility of unsuccessful knockdowns. However, the mortalities after insecticide treatments are different between the water and dsRNA treatments. Although we found no differences in the mortalities among the different dsRNA treatments, there was a significant difference between the two controls (ds*GFP* and water) upon imidacloprid treatment. Mortality was higher in the ds*GFP* treatment than in the water treatment while there was no difference in the control (acetone) mortality. This is intriguing since we found no clear potential target of ds*GFP* in the CPB genome. Differences in mortality between the two controls in RNAi treatments (water and ds*GFP*), although not significantly large, have been observed in other studies (Kishk et al. 2017; Shi et al. 2023). However, it is unclear whether this is simply stochastic or if the ds*GFP* treatment is indeed associated with increased mortality. Higher mortality observed in the ds*GFP* treatment could be due to toxicity caused by the dsRNA molecule itself or any off‐target effects on important genes. There is evidence for ds*GFP* affecting the expression of nontarget genes in honeybees (Jarosch and Moritz 2012; Nunes et al. 2013), as well as hampering development and increasing mortality in the last larval stages in CPB (NN, unpublished data). Further investigation into the possible role of *dsGFP* in increased mortality is warranted.

However, since the control (acetone) mortality is not significantly higher in the ds*GFP* treatment compared to the water treatment in our experiments, it is more plausible that the ds*GFP* treatment enhances the stress caused by the insecticide. As the same result is seen for both *dsLdNA19763* and *dsLdNA20673* treatments, it could also imply that the activation of RNAi machinery, irrespective of the gene, can compound the mortality effect of the insecticide. In other words, RNAi activation itself could make beetles less resistant to insecticides. RNAi is an antiviral immune response that is initiated by dsRNA, which is later broken into smaller RNA fragments (Fire et al. 1998). Small noncoding RNAs, such as microRNA (miRNA), play crucial roles in the regulation of resistance‐related genes and thereby influence resistance in many insects, including CPB (Robles‐Fort et al. 2022; Xiao et al. 2024). RNAi pathways could potentially interfere with existing insecticide resistance pathways and thereby affect resistance levels. Whether there is crosstalk between the two pathways must be investigated by testing more genes and insect populations.

In conclusion, we showed genomic regions that could potentially play a role in resistance against imidacloprid in CPB and attempted to pinpoint key genes of interest. Due to a methodological limitation, our RNAi experiments did not provide clear support for the role of the two candidate genes in imidacloprid resistance. Our study highlights a critical consideration for functional genomics: the potential for the RNAi pathway itself to interact with stress response pathways, which may complicate the validation of genes involved in traits like insecticide resistance. We also emphasize the importance of investigating the genetic and molecular underpinnings of resistance mechanisms in greater detail.

## Author Contributions

**Alitha Edison:** data curation (lead), formal analysis (equal), investigation (lead), methodology (equal), project administration (supporting), resources (equal), software (equal), validation (equal), visualization (lead), writing – original draft (lead), writing – review and editing (equal). **Nijat Nariman:** investigation (equal), methodology (supporting), project administration (supporting), resources (equal), validation (equal), visualization (supporting), writing – review and editing (equal). **Pablo Duchen:** data curation (equal), formal analysis (equal), investigation (supporting), methodology (equal), resources (equal), software (equal), supervision (supporting), validation (equal), visualization (equal), writing – review and editing (equal). **Shuqing Xu:** conceptualization (lead), formal analysis (supporting), funding acquisition (lead), methodology (lead), project administration (lead), resources (equal), supervision (lead), validation (equal), writing – review and editing (equal).

## Conflicts of Interest

The authors declare no conflicts of interest.

## Supporting information


**Figure S1:** Expression profiles of 65 candidate genes from the CPB Gene Expression Atlas. The graph shows the expression profiles of 65 candidate genes in Transcripts Per Million (TPM). The data was downloaded from the CPB GEA website (Wilhelm et al., 2025). The y‐axis indicates the genes in the order of chromosome and position within the chromosome. The panels correspond to the first, second and third instar larvae as indicated on the top. Error bars show standard deviation.
**Figure S2:** Expression profiles of three candidates from the CPB Gene Expression Atlas. The graph shows the expression profiles of three candidate genes (LdNA19763, LdNA19944 and LdNA20673) in Transcripts Per Million (TPM). The data was downloaded from the CPB GEA website (Wilhelm et al., 2025). The colors indicate the three different genes as indicated in the legend. The larval stage is shown on the top. Error bars show standard deviation.
**Table S1:** Primers used for RT‐qPCR.
**Table S2:** dsRNA sequences used for RNAi
**Table S3:** The full list of genes identified in the significant peaks. Genes identified in the genomic regions that are likely to be involved in imidacloprid resistance. The potential function of the gene is given in the ‘BLAST Annotation’ column if available; ‘‐’ indicates lack of information. The ‘Start’ and ‘End’ columns indicate the position of the gene in bp within the specific chromosome.

## Data Availability

All genomic sequences used in this study are deposited in NCBI under the Bioproject PRJNA1121994. All experimental data along with R scripts used to perform statistical tests and generate plots are deposited in Figshare at 10.6084/m9.figshare.28695581.
